# Retrospective analysis of treatment decisions and clinical outcome of Lisfranc injuries: operative vs. conservative treatment

**DOI:** 10.1007/s00264-021-05135-w

**Published:** 2021-08-06

**Authors:** Josefine Graef, Serafeim Tsitsilonis, Marcel Niemann, Tobias Gehlen, Pascal Nadler, Frank Graef

**Affiliations:** 1 Charité - Universitätsmedizin Berlin, Diagnostic and Interventional Radiology and Nuclear Medicine, corporate member of Freie Universität Berlin, Humboldt-Universität zu Berlin, and Berlin Institute of Health, Augustenburger Platz 1, 13353 Berlin, Germany; 2 Charité - Universitätsmedizin Berlin, Center for Musculoskeletal Surgery, corporate member of Freie Universität Berlin, Humboldt-Universität zu Berlin, and Berlin Institute of Health, Augustenburger Platz 1, 13353 Berlin, Germany

**Keywords:** Lisfranc, Tarsometatarsal injury, Distortion trauma

## Abstract

**Purpose:**

Lisfranc injuries are rare and often pose a challenge for surgeons, particularly in initially missed or neglected cases. The evidence on which subtypes of Lisfranc injuries are suitable for conservative treatment or should undergo surgery is low. The aim of this study was to retrospectively analyze treatment decisions of Lisfranc injuries and the clinical outcome of these patients within the last ten years.

**Methods:**

All patients treated due to a Lisfranc injury in a German level I trauma centre from January 2011 until December 2020 were included in this study. Radiologic images and medical data from the patient files were analyzed concerning the classification of injury, specific radiologic variables, such as the Buehren criteria, patient baseline characteristics, and patient outcome reported with the Foot Function Index (FFI).

**Results:**

Ninety-nine patients were included in this study (conservative = 20, operative = 79). The overall clinical outcome assessed by the FFI was good (FFI sum 23.93, SD 24.93); patients that were identified as suitable for conservative treatment did not show inferior functional results. Qualitative radiological factors like the grade of displacement and the trauma mechanism were more strongly associated with the decision for surgical treatment than quantitative radiologic factors such as the distance from the first to the second metatarsal bone.

**Conclusion:**

If the indication for conservative or operative treatment of Lisfranc injuries is determined correctly, the clinical outcome can be comparable. These decisions should be based on several factors including quantitative and qualitative radiologic criteria, as well as the trauma mechanism.

## Introduction

**Fig. 1:**
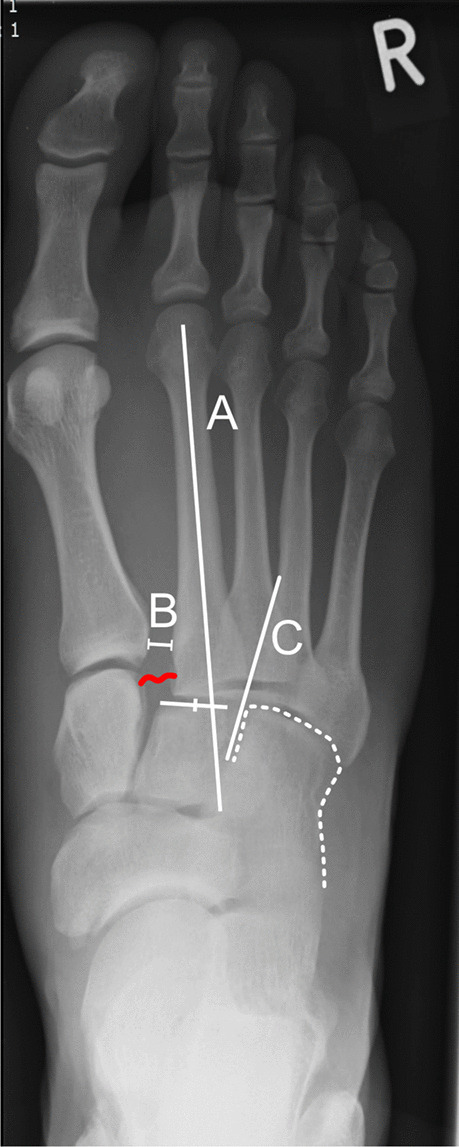
Radiologic criteria indicating if a Lisfranc injury is present in a plain dorsoplantar radiography, as published by Buehren [5]. Buehren A: The shaft axis of the second metatarsal bone physiologically points at the center of the second cuneiform. In this example, the axis does not project at the center, suggesting a Lisfranc injury. Buehren B: The distance of the basis of the first and second metatarsal bone should not exceed 3 mm. In this example, the distance was 7.5 mm. Buehren C: The tangent of the medial basis of the fourth metatarsal bone should exactly be in line with the medial cortex of the cuboid, as seen in this example. The red curved line indicates the position of the Lisfranc ligament between C1 and M2, which is suspected to be torn in this example

The Lisfranc joint line is formed by the tarsometatarsal (TMT) joints and its name was given by French surgeon Jacques Lisfranc de Saint-Martin who performed an amputation of the middle foot at the level of this joint line during the Napoleonic wars [1]. The joint compartments include first cuneiform and first metatarsal bone (C1-M1), first cuneiform and second metatarsal bone (C1-M2), second cuneiform and second metatarsal bone (C2-M2), third cuneiform and third metatarsal bone (C3-M3), cuboid and fourth, and fifth metatarsal bone (Cuboid-M4, Cuboid-M5). The weak link of the joint line is in the region between the bases of M1 and M2, because these two bones are not tightly connected through transverse ligaments. On the other hand, M2-M5 are connected by strong ligaments, which often results in a bony avulsion or rupture of the Lisfranc ligament connecting C1 and M2 (Fig. 1) [2].

Trauma mechanisms leading to an injury of the Lisfranc joint can be subdivided into direct forces (crush injuries) on the foot and indirect injuries (distortion trauma). Indirect injuries can additionally be divided into high-energy trauma (e.g., motor vehicle accidents, fall from height > 3 m) and low-energy trauma (e.g., fall from standing height). Typically, in indirect injuries, the force is either directed along the axis of the foot (e.g., automotive crash with foot on the brake pedal) or the force is directed in a twisted manner around the hindfoot [2].

Lisfranc injuries are rare with a previously reported incidence of 1 in 60,000 new cases per year [3]. More recent studies suggested a rising incidence of 14/100,000 new cases, which could also be the result of increasing availability of high-resolution computer tomography [4]. The process to decide on the correct treatment of Lisfranc injuries can be complex, not least because these injuries are sometimes missed or neglected by patients. The aim of this study was therefore to retrospectively analyze the treatment decisions for all patients with Lisfranc injuries treated in a level I trauma centre within the last ten years to corroborate indications for conservative and operative treatment options. Furthermore, we wanted to report the clinical outcome of these patients considering their grade of injury and type of treatment.

## Methods

All patients who were treated due to an injury of the Lisfranc joint in a single German level I trauma centre from January 2011 until December 2020 were included in this study. The patient cohort was retrospectively selected using patient management software (SAP Business Client 6.5, SAP Walldorf, Germany). The search process was conducted by selecting all patients who were encoded with the ICD-10-GM diagnosis of Lisfranc injuries. This study was approved by the local ethics committee (EA2/025/21).

Medical records of these patients were scanned for specific characteristics (age, sex, trauma mechanism, dates of the trauma, and treatment procedures). X-rays were studied to classify the injuries according to the Hardcastle modification of the classification of Quenu and Kuess [3]. The criteria for diagnosis of Lisfranc injuries as suggested by Buehren were evaluated before and after surgery, too (Fig. 1) [5]. Buehren criteria A and C are qualitative variables, whereas Buehren criterion B is a quantitative measure. X-rays were also analyzed for the presence of an avulsion fracture of the Lisfranc ligament (Fleck sign) [6]. If collateral fractures of the foot and ankle joint adjacent to the Lisfranc joint line were found in the x-rays or CT images, they were reported, too.

Patients were divided into two groups: group A: non-operative treatment and group B: operative treatment. Group B was furthermore subdivided: group B-1: patients who were surgically treated in the acute phase (operation within two weeks after the injury) and group B-2: patients in which operative treatment was delayed beyond two weeks after trauma.

Medical records were also searched for patient-reported outcomes, which are usually assessed with the Foot Function Index (FFI) in our clinic. The FFI is a self-administered two-part score including a pain (FFI pain) and function (FFI function) scale with higher points correlating to worse outcomes (maximum score is 100 points for each of the scales). The FFI is reported for each scale separately and as the sum of both scales (FFI sum) [7].

Statistical analysis was performed using “R” and the software RStudio© (RStudio, Inc., Boston, USA). Results are given as means with standard deviation (SD). Differences between groups were calculated using the Wilcoxon test for non-parametric data; Bonferroni correction was applied for multiple testing. Categorial variables were tested by Fisher’s exact test. Propensity score matching was undertaken to evenly match groups A and B with the “nearest” method.

## Results

The search revealed a total of 100 patients. One patient was excluded; he had to undergo amputation due to a deep wound infection after the patient neglected seeing a doctor for 19 days after a second-degree open Lisfranc injury, leaving 99 patients for study inclusion. Trauma mechanisms and baseline characteristics of both groups of the entire study cohort are displayed in Table 1. The majority of patients sustained a Lisfranc injury after a fall from standing height, of which 16 patients (76.2%) stumbled and the others fell due to a neurological disorder (*n* = 1, 4.8%), a syncope (*n* = 2, 9.5%), during a fistfight (*n* = 1, 4.8%), or slippery ground (*n* = 1, 4.8%). A total of 9 patients (9.1%) sustained the injury doing sports, mainly soccer (*n* = 4, 44.4%); other sports were climbing, surfing, judo, skating, and volleyball (each: *n* = 1, 11.1%). Of the 20 motor vehicle accidents, there are five frontal car crashes (25.0%), 13 motorbike accidents (65.0%), one pedestrian was hit by a car, and another one by a motorbike (each 5.0%). In summary, 36 patients (36.4%) sustained a high-energy trauma.

**Table 1:** Baseline characteristics of operatively and conservatively treated patients with injuries of the Lisfranc joint. Number of collateral fractures is the absolute number of fractured bones of the foot and ankle joint in addition to the Lisfranc injury

	Non-operative	Operative	*p*
*n*	20	79	
Age (median [IQR])	37.95 [15.13]	45.47 [15.84]	0.059
Sex = w (%)	10 (50.0)	32 (40.5)	0.607
Trauma mechanism = *n* (%)			0.850
Bicycle accident	1 (5.0)	3 (3.8)	
Crush injury	1 (5.0)	9 (11.4)	
Fall from height < 3 m	2 (10.0)	7 (8.9)	
Fall from height > 3 m	1 (5.0)	3 (3.8)	
Fall from stairs	2 (10.0)	5 (6.3)	
Fall from standing height	3 (15.0)	18 (22.8)	
Foot stuck	2 (10.0)	2 (2.5)	
Motor vehicle accident	3 (15.0)	17 (21.5)	
Not specified	2 (10.0)	7 (8.9)	
Sports	3 (15.0)	6 (7.6)	
Step in pothole	0 (0.0)	2 (2.5)	
High-energy trauma = *n* (%)	5 (25.0)	31 (39.2)	0.356
Open/closed fracture = *n* (%)			
Closed	20 (100)	73 (92.4)	0.455
III° open	0 (0.0)	6 (7.6)	
Number of collateral fractures	2.50 (1.82)	3.00 (2.29)	0.368

Overall, 79 patients (79.8%) were treated operatively and 20 patients (20.2%) conservatively. Comparison between the conservative and operative group revealed that in those patients treated non-operatively, the majority demonstrated an isolated partial displacement (Hardcastle B2). Homolateral dislocations (Hardcastle type A) and partial or complete divergent dislocations (Hardcastle C) were treated operatively in the majority of all cases. The pre-operative distance between the first and second metatarsal bone (Buehren criterium B) was significantly lower in those patients treated conservatively (Table 2) with a mean diastasis of 1.70 mm (SD 0.83 mm, range 2.6) compared to operatively treated patients with a mean diastasis of 3.61 mm (SD 3.10, range 16.7).

**Table 2:** Comparison between patients treated conservatively and those who underwent surgery concerning the Hardcastle classification as well as the Buehren criteria [3, 5]

	Conservative	Operative	*p*
*n*	20	79	
Hardcastle classification (%)			
A (medial)	1 (5.0)	0 (0.0)	
A (lateral)	4 (20.0)	34 (43.0)	
B1	1 (5.0)	5 (6.3)	
B2 (partial)	10 (50.0)	11 (13.9)	
B2 (complete)	2 (10)	3 (3.8)	
C1 (partial)	2 (10.0)	16 (20.3)	
C2 (complete)	0 (0.0)	10 (12.7)	
Buehren criteria (preoperatively)			
Buehren A = normal (%)	20 (100.0)	16 (20.3)	< 0.001
Buehren B [mm] (mean (sd))	1.70 (0.83)	3.61 (3.08)	0.008
Buehren C = normal (%)	20 (100)	45 (57.0)	0.001
Pre-operative Fleck sign = yes (%)	6 (31.6)	52 (65.8)	0.009

Retrospective analysis of the treatment decision demonstrated that no conservatively treated patient had a positive Buehren B sign (displacement between C1-M2 greater than 3 mm). But due to the fact that homolateral displacements do not affect the M1-M2 distance, the Buehren B criterion cannot be relied on solely. Therefore, we looked at the qualitative Buehren criteria (A and C), too. Here, we could see that if patients had demonstrated a positive Buehren A or C sign, conservative treatment was never chosen. Logistic regression analysis could prove these findings: odd’s ratio (OR) for an operative treatment was distinctly higher for a positive Buehren A criterion (OR 63.45, *p* < 0.001) than for the Buehren B distance (OR 2.09, *p* < 0.05). Odd’s ratio for an operative treatment if patients sustained a high-energy trauma was 6.89 (*p* < 0.05). Receiver operating characteristic (ROC) analysis of the Buehren B distance and the decision for operative or conservative treatment could demonstrate that the cutoff value for operative treatment was 3.0 mm (specificity 95.2%, sensitivity 55.1%, AUC 0.72 (95% CI 0.62–0.82).

For those patients who underwent surgery, the overall time from trauma to primary operative stabilization of the joint line was 31.65 days (80.45 SD, range 436). Fifty-eight patients (74.4%) were operated within two weeks of the injury (group B-1) and the mean time from trauma to primary surgical stabilization was 4.41 days (3.35 SD, range 12). In group B-2 (21 patients, 26.6%), the time span from trauma to operation was 130.12 days (136.65 SD, range 421).

**Fig. 2:**
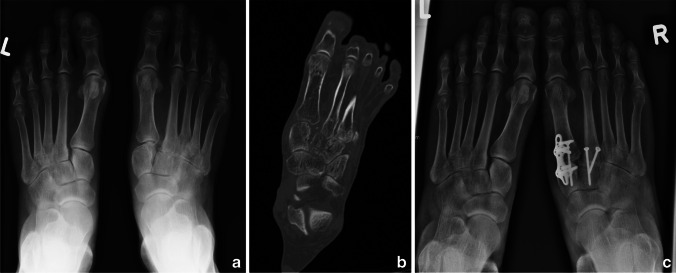
Case of a 49-year-old female patient who was diagnosed a Lisfranc injury of the right foot after a fall from stairs. Conservative treatment was initiated in an external hospital. 5 months later, she visited our clinic with persistent pain. **a** The pre-operative x-ray showed a secondary displacement of the C1-M2 junction; the computer tomography **b** scans could confirm the secondary displacement of the first and second metatarsal bone with a subsequent osteoarthritis. The patient underwent joint fusion of the first and second tarsometatarsal joint (**c**). Seven years after the operation, the clinical outcome was good with a foot function index sum score of 35.2

Five of these patients with surgery after more than 2 weeks (25%) had chronic Lisfranc instabilities with no known time point of trauma. The patients with delay in surgery beyond  weeks were mainly those in which non-operative treatment, initiated in other hospitals, failed (seven patients, 35.0%), or patients who neglected seeing a doctor and visited our clinic due to chronic pain in the aftermath of their injuries (six patients, 30.0%) (Fig. 2). Other reasons were multiple injuries of polytraumatized patients (*n* = 2, 10.0%) who required life-saving operations beforehand, prolonged soft tissue swelling after initial treatment in an external hospital (*n* = 3, 15.0%), or one patient who neglected seeing a doctor due to alcoholism (5.0%).

In group B-1, reduction of the dislocated joint line was performed in 52 cases (89.7%) in an open manner. Closed reduction and temporary K-wire fixation were done in six cases (10.3%). In group B-2, open reduction and internal fixation had to be done in all 20 patients (100%). Comparison of the radiologic results one week after the operation revealed that in group B-2, the Buehren B sign was higher (2.27 mm, 1.69 SD) compared to group B-1 (1.17 mm, 1.29 SD), but these differences were not significant (Table 3). Of 66 patients with temporary K-wire arthrodesis, three patients (4.5%) had to undergo definitive joint fusion one, four and five years after the temporary fixation. All of these three patients had been operated upon within two weeks after the injury. The clinical outcome of patients operated in the acute phase (B-1) and those more than two weeks after the injury (B-2) as assessed by the FFI did statistically not differ between both groups (Table 3).

**Table 3:** Comparison of the radiologic 1 week post-operative results according to the Buehren criteria between those patients operated within two weeks (group A) and those operated on more than two weeks after trauma (group B) [5]

Op within 2 weeks	Yes (group B-1)	No (group B-2)	*p*
*n*	58	21	
Post-operative criteria			
Buehren A = normal (%)	42 (76.4)	14 (70.0)	0.795
Buehren B [mm] (mean (sd))	1.69 (1.29)	2.33 (1.69)	0.089
Buehren C = normal (%)	54 (98.2)	19 (95.0)	1.00
Arthrodesis			< 0.001
Temporary	54 (93.1)	7 (33.3)	
Definitive	1 (1.7)	11 (52.4)	
Both	3 (5.2)	2 (9.5)	
Wound closure			0.083
Primary suture	45 (78.9)	21 (100)	
Secondary suture	4 (7.0)	0 (0)	
Mesh	8 (14.0)	0 (0)	
FFI assessed = yes (%)	18 (31)	14 (70)	
FFI-F	35.30 (30.73)	21.85 (25.03)	0.184
FFI-P	28.31 (22.53)	15.01 (18.40)	0.077
FFI sum	32.22 (26.03)	18.32 (22.87)	0.117

Of all 78 operatively treated patients, 61 patients (78.2%) underwent temporary Lisfranc joint fixation, twelve patients received a definitive arthrodesis (15.4%), and in five patients (6,4%), a combination of temporary fixation and the definitive joint union was performed. Analysis of the fixation methods was conducted for the six compartments of the joint line (C1-M1, C1-M2, C2-M2, C3-M3, Cuboid-M4, Cuboid-M5). In all patients, temporary arthrodesis was performed in a total of 249 joint compartments using K-wires (95.4%) and in 17 compartments (6.5%) using a bridging plate and/or cortex screws. The definitive joint union was achieved in a total of 36 compartments (97.3%) with a locking-compression plate and/or cortex screws, in one case using an additional titan clamp (C1-M1) (SpeedTitan®, DePuy Synthes©) and in another case using the suture-tension device (C1-M2) (TightRope®, Arthrex®).

The FFI was assessed for a total of 43 patients (43.43% of the entire study cohort) with a mean follow-up time of 4.34 years (SD 2.35). The FFI sum of the entire study cohort was 23.93 (SD 24.93), FFI pain = 20.12 (SD 21.18), and FFI function = 27.29 (SD 28.50). In the non-operative treatment group, the FFI was assessed in 10 (50.0%) patients, in the operative group in 33 (41.8%) patients. Comparison between patients with conservative treatment and operative treatment revealed that the clinical outcome assessed by the FFI did not differ statistically between groups. To compare groups A and B with similar baseline characteristics, propensity score matching was performed to match for age, sex, fracture classification, injury mechanism, total number of collateral fractures of the foot and ankle joint, and the follow-up time after the initial treatment. But even after matching, the FFI did still not differ between groups (Table 4).

**Table 4:** Comparison of the clinical outcome assessed by the Foot Function Index for matched groups: operative vs. non-operative group. Groups were matched for age, sex, fracture classification, injury mechanism, total number of collateral fractures of the foot and ankle joint, and the follow-up time after the treatment

	Non-operative	Operative	*p*
FFI assessed = yes (%)	10	10	
Follow-up time [years] = mean (sd)	4.20 (2.04)	4.50 (2.42)	0.768
FFI-F	21.03 (28.46)	30.09 (28.59)	0.487
FFI-P	13.05 (19.35)	24.85 (23.09)	0.231
FFI sum	17.41 (23.85)	27.70 (25.45)	0.363

## Discussion

**Fig. 3:**
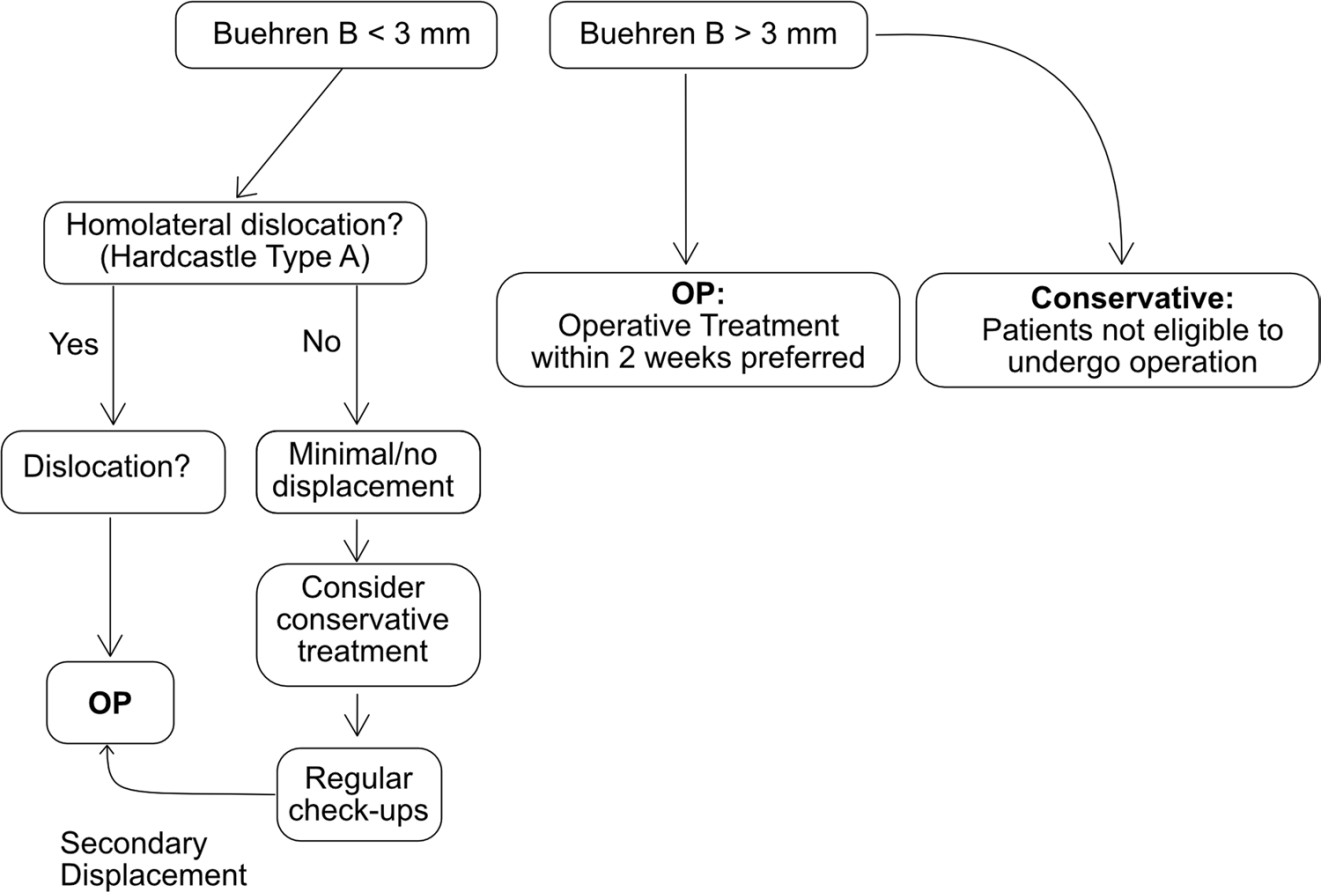
Decision algorithm based on the reported patient cohort in our study. First, in a plain dorsoplantar radiograph of the foot, the Buehren B distance is measured. If it is < 3 mm and if there is no homolateral (Hardcastle type A) injury present, conservative treatment can be considered if there is no or minimal displacement. In cases of a dislocated joint line or multiple tarsal displacements, operative treatment should be favored. If the Buehren B distance is > 3 mm, operative treatment should be favored. Conservative treatment is reserved for patients who are not eligible to undergo operation (e.g., polytraumatized patients with life-threatening injuries)

In this study, it could be demonstrated that if patients were identified as suitable for conservative treatment, they did not show inferior functional results at an average follow-up time of 4.35 years. Based on the findings of this study, we developed a treatment algorithm which could help in the decision process for conservative or operative treatment of Lisfranc injuries (Fig. 3).

The decisions to treat Lisfranc injuries either conservatively or operatively are controversially debated. Nunley et al. reported a classification system for Lisfranc injuries in athletes after midfoot sprains [8]. In their study, all patients with a diastasis between M1 and M2 greater than 2 mm underwent an operation and those patients with a distance less than 2 mm were treated conservatively. The clinical outcome for this decision protocol was equally good with excellent outcomes for both the operative and conservative groups.

In the present study, too, the distance between M1 and M2 was significantly lower in the conservative group compared to the operative group. But on the other hand, it was not used as the sole indicator for the treatment decision. Retrospective analysis of the treatment options showed that the combination of two positive Buehren criteria was strongly associated with the decision for an operative procedure. Furthermore, logistic regression analysis demonstrated that qualitative parameters, such as the Buehren A criterion had a higher impact on the decision to treat operatively than the sole absolute distance between M1 and M2. The reason not to rely on this distance solely is also underscored by the fact that a high percentage of injuries are homolateral dislocations (Quenu and Kuess type A), which affect the entire joint line.

Similar to the present study, the clinical outcome after conservative vs. operative treatment of minimally displaced Lisfranc injuries has recently been reported to be equally good for both treatment groups [9]. Although in our study, injuries with high and low displacement grades were included likewise, the clinical outcome was rendered comparable by propensity score matching of both treatment groups. In the study from Chen et al., a high secondary diastasis rate of 54% of conservatively treated Lisfranc injuries was reported, and all of those required surgical stabilization. Ren et al. recently reported a secondary diastasis rate of 34.1% of conservatively treated non-displaced subtle ligamentous Lisfranc injuries. In their study, patients with non-displaced Lisfranc injuries—who underwent percutaneous fixation—had significantly better clinical outcome measures and less complication rates compared to conservatively treated patients [10]. However, there is no clear recommendation to operatively stabilize non-displaced Lisfranc injuries immediately, as subsequent surgical management in these secondary dislocations has been shown to result in outcomes which were comparable to those injuries which remained minimally displaced, if treated in a timely manner [9].

In accordance with our study, the clinical outcome reported using the FFI did not differ between those patients who were operated within two weeks after the injury and those patients who were operated on more than two weeks after trauma. But, the results suggested that those patients who were operated upon more than two weeks after the injury had a slightly better outcome (Table 3). This could be due to the fact that in those patients, primary arthrodesis was performed in the majority of all cases and in group B-1; temporary arthrodesis was done in 93.1% of all cases. Naturally, temporary arthrodesis procedures using K-wires or screws damage the cartilage of joints, which ultimately can lead to osteoarthritis. On the other hand, definitive tarsometatarsal joint fusions can alter the biomechanics of the entire foot arch during gait and stance and lead to higher peak pressures on the forefoot [11]. This could, ultimately, lead to subsequent osteoarthritis in neighboring joints. In the end, the decision to perform a temporary arthrodesis or definitive joint fusion should take into account the patient’s mobility, age, time point of trauma, and the grade of cartilage damage with additional intraarticular tarsometatarsal fractures.

One way to avoid additional damage to the joints’ cartilage by K-wires or screws is by temporary joint stabilization using a bridging plate. It could already be demonstrated that this method resulted in better functional and radiological outcomes compared with transarticular screws [12]. The disadvantages of this procedure could be a higher soft tissue dissection. In this study, merely four temporary joint fusions were done using a bridging plate and in two cases using a combination of a bridging plate and K-wires or screws. Therefore, a statistical analysis on the clinical outcome after different temporary stabilization methods was not performed here.

The main limitation of this study was the selection bias since 21 conservatively and 78 operatively treated patients were included. This problem was tackled by propensity score matching to generate two similar groups. But due to the retrospective nature of this study, even the best matching method is limited by the depth of detail documented in the medical records. In a future prospective study, pairwise matching should be undertaken to take into account patient characteristics such as age, sex, and chronic diseases and also the trauma mechanism and grade of dislocation of the Lisfranc joint. Furthermore, in a future study, case matching should consider different fixation methods for definitive and temporary stabilization procedures.

## Conclusion

The decision to treat Lisfranc injuries operatively or conservatively should always include qualitative parameters such as the grade of displacement (Buehren criteria A and C) and quantitative variables like the M1-M2 distance (Buehren criterion B) but also take into account the trauma mechanism. If conservative treatment is chosen, regular checkups are required to not miss secondary displacements.

## Data Availability

Data are not available in a public repository.
